# Development and Preliminary Evaluation of a Nanoparticle-Assisted PCR Assay for the Detection of *Cryptosporidium parvum* in Calves

**DOI:** 10.3390/ani12151953

**Published:** 2022-08-01

**Authors:** Qian Yao, Xin Yang, Yi Wang, Junwei Wang, Shuang Huang, Junke Song, Guanghui Zhao

**Affiliations:** 1 College of Veterinary Medicine, Northwest A&F University, Xianyang 712100, China; yaoqian@nwafu.edu.cn (Q.Y.); xinyang@nwafu.edu.cn (X.Y.); wangyi9614@163.com (Y.W.); wjunwei@nwafu.edu.cn (J.W.); huangshuang7892021@163.com (S.H.); sjk7998@163.com (J.S.); 2 State Key Laboratory of Veterinary Etiological Biology, Key Laboratory of Veterinary Parasitology of Gansu Province, Lanzhou Veterinary Research Institute, Chinese Academy of Agricultural Sciences, Lanzhou 730046, China

**Keywords:** *Cryptosporidium parvum*, nanoparticle-assisted PCR, *cgd3_330*, calves

## Abstract

**Simple Summary:**

Accurate and rapid detection of *Cryptosporidium parvum* is useful for the prevention and control of cryptosporidiosis in humans and animals. This study developed a nano-PCR assay for the rapid detection of *C. parvum* in calves for the first time, and it was ten-fold more sensitive than the normal PCR assay and had no cross-reaction with other common gastrointestinal pathogens. Further analyses of faecal samples of calves indicated potential usage of the nano-PCR assay in clinical settings.

**Abstract:**

*C. parvum* is an important diarrheal pathogen in humans and animals, especially in young hosts. To accurately and rapidly detect *C. parvum* infection in calves, we established a nano-PCR assay targeting the *cgd3_330* gene for the specific detection of *C. parvum*. This nano-PCR assay was ten times more sensitive than that of the normal PCR assay by applying the same primers and did not cross-react with *C. andersoni*, *C. bovis*, *C. ryanae*, *Balantidium coli*, *Enterocytozoon bieneusi*, *Giardia lamblia*, and *Blastocystis* sp. To further test the nano-PCR in clinical settings, a total of 20 faecal samples from calves were examined by using the nano-PCR, the normal PCR, and the nested PCR assays. The positive rates were 30% (6/20), 30% (6/20), and 25% (5/20) for the nano-PCR, the normal PCR, and the nested PCR assays, respectively, indicating that the nano-PCR and the normal PCR assays had the same positive rate (30%). Taken together, the present study could provide a candidate method for the specific detection of *C. parvum* infection in calves in clinical settings.

## 1. Introduction

Bovine cryptosporidiosis is an important disease caused by the zoonotic protozoan *Cryptosporidium* spp., with the clinical syndromes of diarrhea, fever, and massive fluid loss in the gastrointestinal tracts, especially in young animals [1]. Although adult cattle infected with *Cryptosporidium* spp. usually exhibit asymptomatic shedding of oocysts, calves often suffer severely fatal diarrhea [2]. Of over 40 valid *Cryptosporidium* species reported, four species are commonly detected in cattle, namely *C. parvum*, *C. andersoni*, *C. ryanae,* and *C. bovis*. *Cryptosporidium parvum* was found to be predominant in calves of the suckling period, *C. andersoni* was mostly found in adult and yearling cattle, while *C. bovis* and *C. ryanae* were usually detected in post-weaned calves [2,3,4,5,6]. Among them, *C. parvum* has been identified as the major pathogenic and zoonotic pathogen, endangering the health of humans and the development of the breeding industry [7]. However, there have been no effective vaccines or drugs for the prevention and control of *C. parvum* infection or cryptosporidiosis. 

Accurate and rapid diagnosis is of great significance to the prevention and control of parasitic diseases. To date, several methods for detecting *C. parvum* infection have been developed, including microscopic examination, immunological assays, and nucleic acid-based detection methods. Of them, traditional microscopic examination is the most common technique to detect *C. parvum* oocysts, which are easily recognized in fresh stools by microscopic examination [8]. However, this method is time-consuming, lacks sensitivity and requires a well-trained examiner. Immunological methods have several advantages over microscopic examination in terms of sensitivity and specificity. In some western countries, there are several commercially available kits for detecting *C. parvum*, including enzyme immunoassay, immunofluorescence assay, and immune-chromatography test formats [9]. However, this approach is easily contaminated, and costly. Nucleic acid-based detection methods have been widely used for the detection of *C. parvum* with high sensitivity and specificity. For example, a nested polymerase chain reaction (nested PCR) targeting the *gp60* gene can effectively identify *C. parvum* and its subtypes [10,11,12]. However, nested PCR can increase the operational complexity as well as susceptibility to contamination [13]. Thus, it is urgently needed to establish a rapid, user-friendly, and reliable assay to detect *C. parvum* infection.

With the development of nanotechnology, nanoparticles, including carbon nanotubes, silver nanoparticles, titanium oxide nanoparticles, zinc oxide nanoparticles, quantum dots, and gold nanoparticles (AuNPs), have been used in almost all fields of science, and nano-PCR has become a novel type of PCR technique and is more sensitive than normal PCR [14]. Previous studies have shown that nanoparticles could bind to single-strand DNA and play a similar role to the single-strand binding protein, significantly reducing mismatches between primers and templates, improving the specificity of the PCR reaction, and effectively reducing non-specific amplification [15,16]. Furthermore, using AuNPs as a PCR additive could increase detection sensitivity by 5- to 10-fold in standard PCR [17]. Recently, some nano-PCR methods have been applied for the detection of viruses, bacteria, and parasites [18,19,20,21,22,23]. Previously, one nano-PCR was established for the detection of *Cryptosporidium* infection in several animals. This nano-PCR was found to be sensitive and specific and had potential application in the detection of *Cryptosporidium* infection in clinical settings [22]. However, it could not distinguish between those pathogenic *Cryptosporidium* species, especially for *C. parvum*, an important diarrheal pathogen threatening the health of humans and animals. Therefore, we developed and assessed a sensitive and species-specific nano-PCR diagnostic method for the detection of *C. parvum* infection in this study.

## 2. Materials and Methods

### 2.1. Parasites, Clinical Samples, and gDNA Samples

In this study, the oocysts of *C. parvum* were purified, isolated, and preserved in our lab. Clinical faecal samples of calves were previously collected from a dairy cattle farm in Yangling, Shaanxi province, and stored in our laboratory at 4 °C. Genomic DNA (gDNA) samples were isolated from oocysts of *C. parvum* and clinical faecal samples using a commercial kit as described [24]. Those gDNA samples for *C. andersoni*, *C. bovis*, *C. ryanae*, *Balantidium coli*, *Enterocytozoon bieneusi*, *Blastocystis* sp., and *Giardia lamblia* were from previous studies [25,26,27,28].

### 2.2. PCR Primers

Through comparative genomic analysis, a gene, namely *cgd3_330*, would have the potential to differentiate *C. parvum* from the remaining three common *Cryptosporidium* species (*C. andersoni*, *C. ryanae,* and *C. bovis*) in cattle. Two specific primers (Table 1) based on the *cgd3_330* gene of *C. parvum* for the normal PCR and the nano-PCR were designed and synthesized as reported [22].

### 2.3. Nested PCR

Nested PCR was used to detect *C. parvum* infection in faecal samples based on the reported primers as shown in Table 1 [10]. The protocol of nested PCR to amplify the *gp60* gene was the same as in the previous report [10].

### 2.4. Optimization of the Normal PCR Assay for C. parvum

The normal PCR to obtain the *cgd3_330* gene was conducted in a 12 μL reaction mixture. The initial PCR reaction system and conditions were the same as in the previous study [22]. On this basis, we optimized the annealing temperature and the concentration of MgCl_2_. The annealing temperatures ranged from 50 °C to 60 °C and the concentration of MgCl_2_ ranged from 0.42 mM to 2.92 mM. All the products were verified under a UV transilluminator after electrophoresis.

### 2.5. Optimization of the Nano-PCR Assay for C. parvum

Nano-PCR reaction system was carried out in a 12 μL reaction mixture containing 6 μL 2 × Nano-QPCR buffer plus AuNPs (Catalog no. NHS20-3; Shanghai Hushi Medicine Technology Co., Ltd., Shanghai, China), 10 pmol each primer, 1 U *Taq* enzyme mix (Shanghai Hushi Medicine Technology Co., Ltd., Shanghai, China), and 1 μL gDNA template. The initial reaction condition was also the same as in the previous study [22]. The annealing temperature (50–60 °C) and primer amounts (2–14 pmol) were then optimized. All the products were visualized under a UV transilluminator after 1% agarose gel electrophoresis.

### 2.6. Sensitivities of the Normal PCR and the Nano-PCR Assays

Ten-fold serial dilutions of one gDNA sample of *C. parvum* (102 ng/μL) were used to analyze the sensitivities of the normal PCR and the nano-PCR assays, with ddH_2_O used as a negative control. All the products were verified using a UV transilluminator after electrophoresis.

### 2.7. Specificities of the Normal PCR and the Nano-PCR Assays

The gDNA samples of *C. parvum* (102 ng/μL), *C. andersoni* (29 ng/μL), *C. bovis* (35 ng/μL), *C. ryanae* (32 ng/μL), *B. coli* (24 ng/μL), *E. bieneusi* (31 ng/μL), *G. lamblia* (18 ng/μL), and *Blastocystis* sp. (23 ng/μL) were used to verify the specificities of the established detection methods.

### 2.8. Assessment of the Nano-PCR in Faecal Samples

Twenty faecal samples were used to assess the accuracy of the nano-PCR assay. Meanwhile, the nested PCR and the normal PCR were performed on these samples for comparison, respectively. The representative products were sent for sequencing (Sangon Biotech (Shanghai) Co., Ltd., Shanghai, China).

## 3. Results

### 3.1. Optimization of the Normal PCR and the Nano-PCR for C. parvum

**Figure 1:**
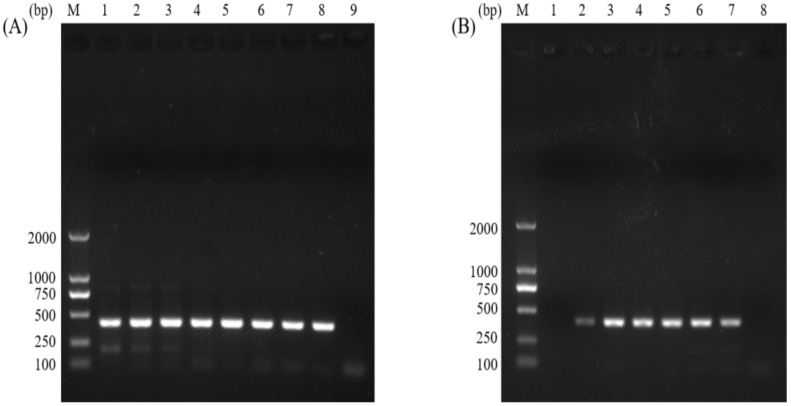
Optimization of the annealing temperature (**A**) and the concentration of MgCl_2_ (**B**) of the normal PCR assay. (**A**) lane M: DL2000 DNA Marker (TakaRa); lane 1: 50 °C; lane 2: 50.5 °C; lane 3: 52 °C; lane 4: 54 °C; lane 5: 56 °C; lane 6: 58 °C; lane 7: 59.5 °C; lane 8: 60 °C; lane 9: negative control. (**B**) lane M: DL2000 DNA Marker (TakaRa); lane 1: 0.42 mM; lane 2: 0.83 mM; lane 3: 1.25 mM; lane 4: 1.67 mM; lane 5: 2.08 mM; lane 6: 2.5 mM; lane 7: 2.92 mM; lane 8: negative control.

**Figure 2:**
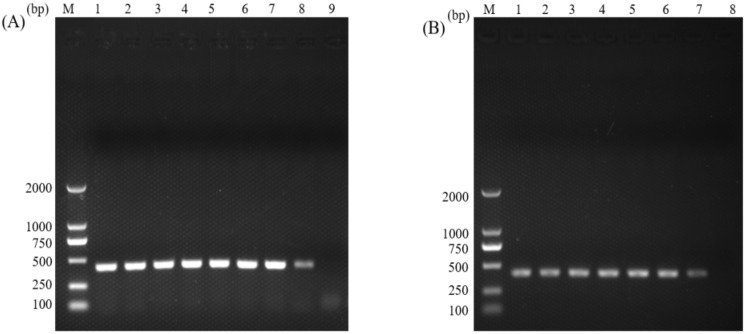
Optimization of the annealing temperature (**A**) and the primer amounts (**B**) of the nano-PCR assay. (**A**) lane M: DL2000 DNA Marker (TakaRa); lane 1: 50 °C; lane 2: 50.5 °C; lane 3: 52 °C; lane 4: 54 °C; lane 5: 56 °C; lane 6: 58 °C; lane 7: 59.5 °C; lane 8: 60 °C; lane 9: negative control. (**B**) lane M: DL2000 DNA Marker (TakaRa); lane 1: 2 pmol; lane 2: 4 pmol; lane 3: 6 pmol; lane 4: 8 pmol; lane 5: 10 pmol; lane 6: 12 pmol; lane 7: 14 pmol; lane 8: negative control.

The gDNA samples from *C. parvum* oocysts were used as templates in the normal PCR and the nano-PCR assays, and the target fragment was about 410 bp in length. The optimal annealing temperature for normal PCR was 56 °C (Figure 1A), and the concentration of MgCl_2_ was 1.25 mM (Figure 1B). For nano-PCR, the optimal annealing temperature was 56 °C (Figure 2A), and the concentration of primers was 10 pmol (Figure 2B).

### 3.2. Sensitivities of the Normal PCR and the Nano-PCR Assays

**Figure 3:**
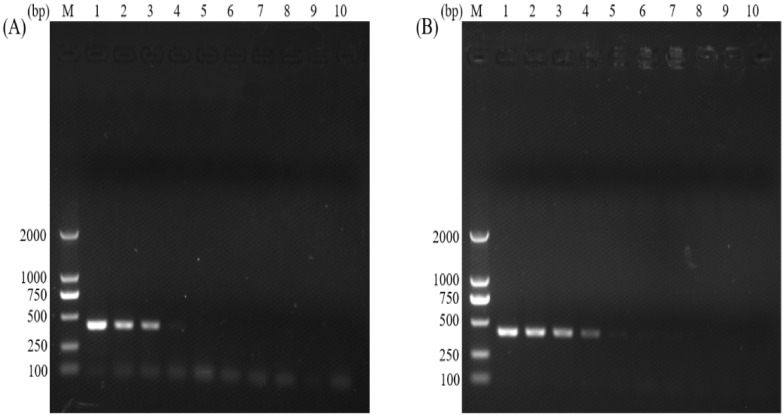
A serial ten-fold dilution of gDNA samples from *C. parvum* oocysts was used to analyze sensitivities of the normal PCR (**A**) and the nano-PCR (**B**) assays. Lane M: DL2000 DNA Marker (TakaRa); lane 1: 102 ng; lane 2: 10.2 ng; lane 3: 1.02 ng; lane 4: 102 pg; lane 5: 10.2 pg; lane 6: 1.02 pg; lane 7: 102 fg; lane 8: 10.2 fg; lane 9: 1.02 fg; lane 10: negative control.

The sensitivities of the normal PCR and the nano-PCR assays were analyzed by the ten-fold serial dilutions of one gDNA sample of *C. parvum*. The results showed that the nano-PCR was ten-fold more sensitive than the normal PCR, with a detection limit of 1.02 ng and 102 pg for the normal PCR (Figure 3A) and the nano-PCR (Figure 3B), respectively.

### 3.3. Specificities of the Normal PCR and the Nano-PCR Assays

**Figure 4:**
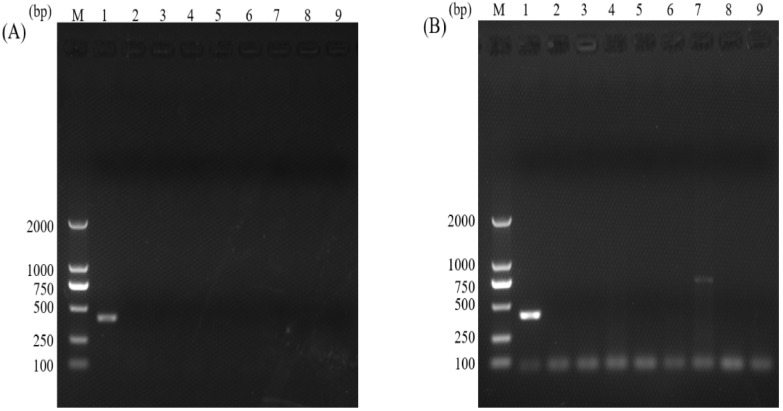
Specificities of the nano-PCR (**A**) and the normal PCR (**B**) assays. Lane M: DL2000 DNA Marker (TaKaRa); lane 1: gDNA sample of *C. parvum*; lane 2: gDNA sample of *C. andersoni*; lane 3: gDNA sample of *C. bovis*; lane 4: gDNA sample of *C. ryanae*; lane 5: gDNA sample of *Blastocystis* sp.; lane 6: gDNA sample of *G. lamblia*; lane 7: gDNA sample of *E. bieneusi*; lane 8: gDNA sample of *B. coli*; lane 9: negative control.

The specificities of the normal PCR and the nano-PCR assays were assessed by testing the gDNA samples of eight intestinal pathogens. In the nano-PCR, the specific band was only found in a gDNA sample of *C. parvum* (Figure 4A). Similarly, normal PCR could amplify the target band in the gDNA sample of *C. parvum*. Although a gDNA sample of *E. bieneusi* could also be amplified, this non-specific band had a larger size than the specific band of *C. parvum* so that these two species could be identified in the normal PCR (Figure 4B). The results showed that both the normal PCR and the nano-PCR had good specificity in detecting *C. parvum*.

### 3.4. Assessment of the Nano-PCR for C. parvum in Clinical Settings

**Figure 5:**
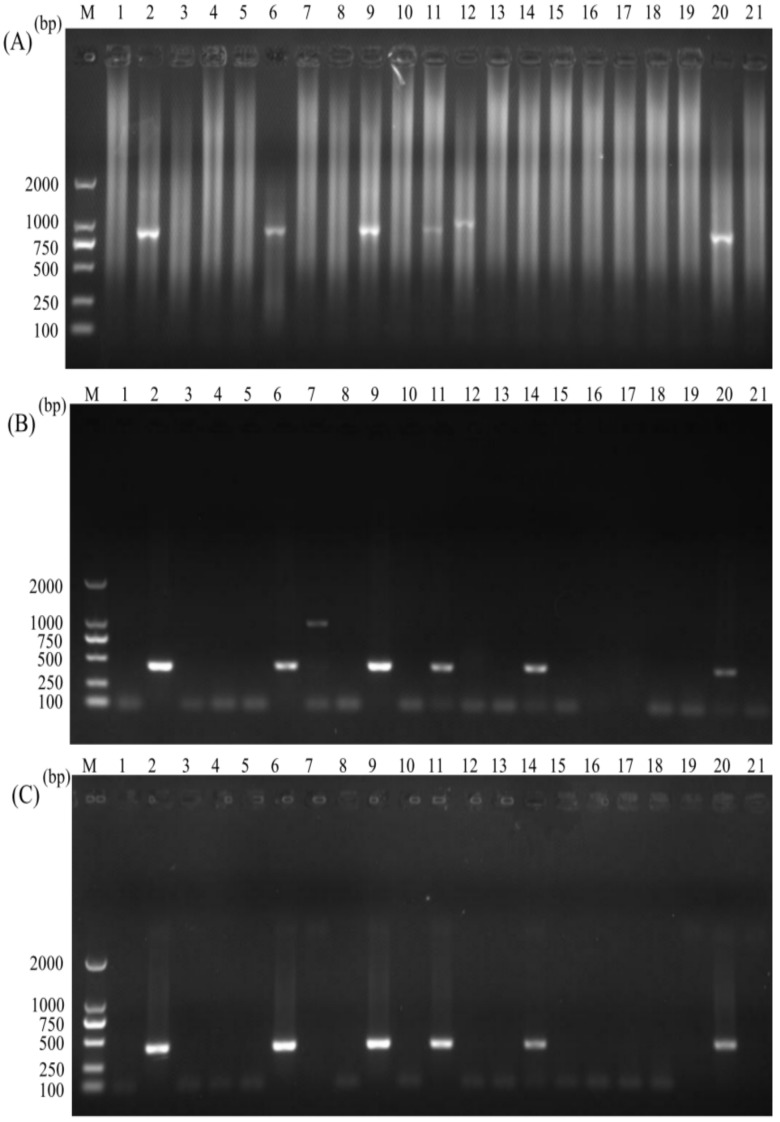
Detection of *C. parvum* in clinical samples by using the nested PCR (**A**), the normal PCR (**B**) and the nano-PCR assays (**C**). Lane M: DL2000 DNA Marker (TaKaRa); lanes 1–20: faecal samples of calves; lane 21: negative control.

Twenty clinical samples of calves were determined for infection of *C. parvum* using nested PCR, normal PCR, and nano-PCR, respectively. Among those clinical samples, 25% (5/20), 30% (6/20), and 30% (6/20) were positive for *C. parvum* infection by using the nested PCR (Figure 5A), the normal PCR (Figure 5B), and the nano-PCR (Figure 5C). The sample in the lane 12 in the nested PCR was likely infected with other *Cryptosporidium* species but not *C. parvum* since the band was larger than that of other positive samples. The sample in the lane 7 in the normal PCR might be infected with other pathogens but not *C. parvum* since the band was nearly two-fold larger than that of other positive samples, and we have tried to sequence the amplicon but failed. The amplicon in the lane 14 was negative in the nested PCR but positive in the normal PCR and the nano-PCR was sent to Sangon Biotech for sequencing. The sequence was identified to be *C. parvum* for the amplicon in the lane 14 in the nano-PCR.

## 4. Discussion

Limitations of microscopic and immunological techniques resulted in the involvement of nucleic acid-based detection methods (e.g., PCR, nested PCR, qPCR, ddPCR, and LAMP) for the detection of *C. parvum* [29,30,31]. Compared to microscopic examination and immunological assays, the molecular techniques were found to be more sensitive and specific [32]. The nested PCR based on the *gp60* locus has been widely used for detecting and subtyping *Cryptosporidium*, but it is time-consuming, easily contaminated, and needs to be combined with RFLP or sequencing for detection of *C. parvum* [33,34]. The qPCR assay was sensitive, specific, and reproducible for detecting *C. parvum* and significantly improved laboratory workflow and turnaround times, but it was dependent on the accuracy of the standard curve and required expensive instruments [35,36]. The ddPCR was an emerging method for detecting *Cryptosporidium* infection, which provided absolute quantitation without the use of calibration curves [37]. However, the template copy numbers of ddPCR were less than those of qPCR, and the average cost per sample was nearly two times higher than that of qPCR [37]. LAMP was an accurate, rapid, and sensitive method used for detecting *Cryptosporidium* species, but it was easily polluted by aerosol, which commonly exists in the environment [38,39]. In the present study, a novel, rapid, and sensitive nano-PCR assay was developed for the specific detection of *C. parvum* in faecal samples of calves.

Several useful molecular markers, e.g., *18S rRNA* [40] and *gp60* [12] genes, have been widely applied in the accurate detection of *Cryptosporidium* infection, significantly contributing to the development of *Cryptosporidium* infection and cryptosporidiosis molecular epidemiology. Previously, we compared the nano-PCR, the normal PCR, and the nested PCR for detecting *Cryptosporidium* infection based on the *18S rRNA* gene. The results indicated that the nano-PCR was 100-fold more sensitive than the normal PCR, and the nano-PCR showed a higher detection rate than the nested PCR, reflecting the potential superiority of the nano-PCR in the detection of *Cryptosporidium* infection in clinical settings compared with the nested PCR [22]. However, due to the high conservatism of these gene loci among *Cryptosporidium* species, detection methods applying these loci are usually unable to effectively identify *Cryptosporidium* at the species level. Through comparative genomic analysis, the *cgd3_330* gene was recognized as a potential marker for the detection of *C. parvum* in cattle, and no homologous gene sequences of the *cgd3_330* gene were found for the other three *Cryptosporidium* species (*C. bovis*, *C. ryanae,* and *C. andersoni*) commonly found in cattle based on results from BLASTN and BLAST analyses in CryptoDB and NCBI, respectively. Thus, we used the *cgd3_330* gene of *C. parvum* in a nano-PCR assay for accurate detection of *C. parvum* in faecal samples of calves.

The developed nano-PCR method in the present study was ten-fold more sensitive than the normal PCR, with a detection rate of 30% (6/20). Meanwhile, this method was specific for detection of *C. parvum* infection, and no cross-reaction was found with the other seven common pathogens in the faecal samples of calves, including *C. ryanae*, *C. andersoni,* and *C. bovis*. We have sequenced the *18S rRNA* gene of the three *Cryptosporidium* species negative for amplification of the *cgd3_330* gene of *C. parvum*. They were identified to be *C. bovis*, *C. ryanae,* and *C. andersoni*, respectively, by BLAST analyses and phylogenetic analyses (Supplementary Materials: Figure S1). Meanwhile, we also used the *gp60* locus, a common recognized genetic marker for zoonotic *Cryptosporidium* species, in detecting clinical samples, and samples positive for both markers were all identified to be *C. parvum*. Interestingly, a clinical sample in the lane 14 that was negative for amplification of the *gp60* gene was also positive in the nano-PCR based on the *cgd3_330 gene*, and sequencing verified that this sample was infected with *C. parvum*. Taking these together, the nano-PCR targeting the *cgd3_330* gene could detect more positive samples than the nested PCR targeting the *gp60* gene but needs to be verified in future studies.

Recently, the outbreaks of diarrhea caused by *C. parvum* have led to hundreds of deaths in calves on several farms in China, causing great economic losses to the farms [41,42]. With the application of the nano-PCR, we could rapidly identify *C. parvum* infection early, and the targeted interventions, such as isolating and treating infected animals, and sterilizing the environment, could be conducted to block the transmission of *C. parvum* among animals and minimize animal death and economic losses.

## 5. Conclusions

The present study established an efficient nano-PCR assay targeting the *cgd3-330* gene for specific detection of *C. parvum* in calves, and it could effectively detect *C. parvum* in a small scale of clinical samples. This nano-PCR assay showed superiority in detection of *C. parvum* infection compared with the normal PCR and the nested PCR techniques. Further studies are needed to test the nano-PCR on large-scale clinical samples from diverse hosts and geographical areas.

## Figures and Tables

**Table 1:** Sequences of the primers used in this study.

PCR Assays	Primer Names	Sequences (5′–3′)	Product Sizes (bp)
Normal PCR /Nano-PCR	*cgd3_330*-F	AGTGGTTACAGGTGGGATGAGT	~413
	*cgd3_330*-R	GCGAGTTTCCTTGATTCATAGC	
Nested PCR	*gp60*-F1	TTACTCTCCGTTATAGTCTCC	~915
	*gp60*-R1	GGAAGGAACGATGTATCTGA	
	*gp60*-F2	TCCGCTGTATTCTCAGCC	~800
	*gp60*-R2	GCAGAGGAACCAGCATC	

## Data Availability

Data is contained within the article.

## Supplementary Materials

The following are available online at https://www.mdpi.com/article/10.3390/ani12151953/s1. Figure S1: Phylogenetic tree generated by the Neighbor-Joining method using partial sequences of the *18**S* *rRNA* gene of four *Cryptosporidium* species in the present study and representative sequences of other *Cryptosporidium* species available in the GenBank^TM^. The sequences obtained in this study are marked with red triangles. Bootstrap values (>70) are indicated at the nodes. Scale bar indicates 0.02 nucleotide substitutions/site.
