# Supplementary figures and images for: Development and Preliminary Evaluation of a Nanoparticle-Assisted PCR Assay for the Detection of Cryptosporidium parvum in Calves
Source: Animals (Basel). 2022 Aug 1;12(15):1953. doi: 10.3390/ani12151953 (PMC9367258; doi:10.3390/ani12151953)

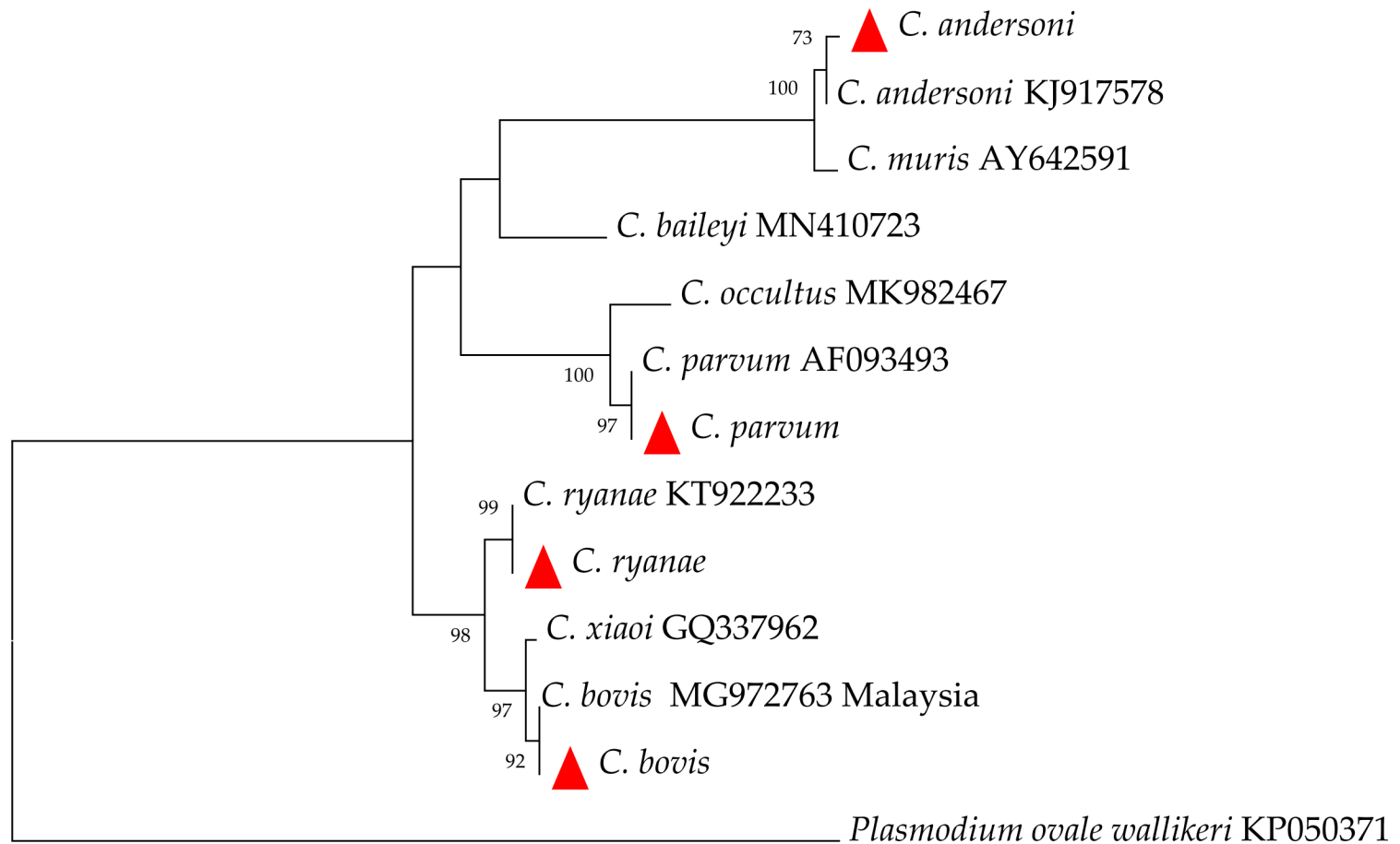

Supplement: Supplementary file 1 [file animals-12-01953-s001.zip › animals-1770101-Figure S1.pdf]
